# Role of Religious Leaders in Promoting Contraceptive Use in Nigeria: Evidence From the Nigerian Urban Reproductive Health Initiative

**DOI:** 10.9745/GHSP-D-18-00135

**Published:** 2018-10-03

**Authors:** Sunday A. Adedini, Stella Babalola, Charity Ibeawuchi, Olukunle Omotoso, Akinsewa Akiode, Mojisola Odeku

**Affiliations:** a Demography and Social Statistics Department, Faculty of Social Sciences, Obafemi Awolowo University, Ile-Ife, Nigeria; and Demography and Population Studies Programme, Schools of Public Health and Social Sciences, University of the Witwatersrand, Johannesburg, South Africa.; b Johns Hopkins Center for Communication Programs, Baltimore, MD, USA.; c Nigerian Urban Reproductive Health Initiative, Abuja, Nigeria.

## Abstract

**Background:**

Despite the many supply- and demand-side interventions aimed at increasing contraceptive uptake, the modern contraceptive prevalence rate in Nigeria has remained very low (9.8%). Religion is an important part of the sociocultural fabric of many communities. As such, religious leaders have the power to inhibit or facilitate effective adoption of contraceptive methods to support family health. We assess the association of exposure to religious leaders' tailored scriptural family planning messages with contraceptive use in Nigeria.

**Methods:**

This cross-sectional study used data from a Measurement, Learning and Evaluation Project survey conducted in 2015 in 4 Nigerian states—Federal Capital Territory, Kaduna, Kwara, and Oyo. The final study sample was restricted to 9,725 non-pregnant women aged 15 to 49 years. Data analysis included descriptive statistics and binary logistic regression analysis to explore significant relationships between current use of a modern contraceptive method, exposure to family planning messages from religious leaders, and selected background characteristics.

**Results:**

About 2 in 5 women reported being exposed to family planning messages from religious leaders in the past year. Bivariate results revealed a higher uptake of modern contraceptives among women with high exposure to different NURHI interventions (35.5%) compared with respondents in the low or medium exposure categories (14.5% and 24.5%, respectively). The multivariable analysis revealed significantly higher contraceptive uptake among women who had exposure to family planning messages from religious leaders relative to those with no exposure (odds ratio=1.70; 95% confidence interval, 1.54 to 1.87; *P*<.001). This association remained significant after adjustment for background characteristics and other selected variables.

**Conclusion:**

Interventions that engage clerics of different faiths as change agents for shaping norms and informing behaviors about family planning and contraceptive use are crucial for increasing contraceptive uptake in Nigeria.

## Abstract (precis)

Exposure to family planning messages from religious leaders was significantly associated with higher modern contraceptive use, after accounting for background characteristics and other variables such as myths and misconceptions. Engaging religious leaders to support positive social norms is an important strategy to improving voluntary contraceptive use in Nigeria.

## INTRODUCTION

Despite the many supply- and demand-side interventions aimed at increasing contraceptive uptake, modern contraceptive prevalence rate (mCPR) has remained very low in Nigeria. Nigeria's mCPR is one of the lowest globally, currently estimated at 9.8%.^1^ In comparison, the mCPRs of other sub-Saharan African countries, such as Rwanda and Malawi, are much higher (45% and 62%, respectively).^2^^,^^3^

The health and socioeconomic benefits of contraceptive use have been well-documented. These benefits include improved quality of life, increased well-being of families and communities, improved maternal and newborn health outcomes,^4^^–^^7^ reduced poverty, increased female education,^8^ and additional noncontraceptive health benefits of hormonal methods.^9^

In contrast, unplanned families face enormous health and developmental challenges. The micro- and macro-level socioeconomic and health consequences of high fertility—defined as a total fertility rate of 5 or more children born per woman—are diverse. At the micro or household level, children and women in high-fertility families are predisposed to tremendous health risks, particularly high childhood and maternal morbidity and mortality.^10^^–^^13^ At the macro or national level, high fertility has been shown to slow down socioeconomic growth, diminish human capital investment, and aggravate environmental threats and degradations.^14^

While Nigeria's socioeconomic situation clearly shows that the country trails other countries in many indicators of development, an expanded family planning program could help reduce barriers to development. For example, researchers have suggested that several supply- and demand-side factors may explain the low level of contraceptive prevalence in Nigeria. These studies have implicated such factors as age at sexual debut, educational attainment, fertility intentions, and household wealth as important predictors of contraceptive use.^15^^,^^16^ Other commonly cited factors inhibiting the success of family planning program in Nigeria include cultural beliefs,^17^ fear of adverse effects, religious prohibition,^18^ partner disapproval, poverty,^19^ and common myths and misconceptions.^20^^–^^22^

Of these factors, the role of religious leaders in facilitating or inhibiting contraceptive uptake has been less well explored. Religion is often an important part of the cultural fabric of communities and, as such, can influence decision making, ideologies, and moral and ethical behaviors.^23^ Religious beliefs on issues of fertility, contraceptive adoption, and abortion can differ greatly among Protestant Christians, Catholics, Muslims, and traditionalists. For instance, abortion is generally considered forbidden in Islam, although most schools of thought allow for early abortion (defined as the first 40, 90, or 120 days of pregnancy, depending on the school of thought) and for abortion in certain circumstances such as when the mother's life is in danger.^24^ The Catholic Church allows only natural methods of contraception.^23^ Many religious leaders hold beliefs that lead them to speak against modern contraceptive methods. As a result, they can greatly influence the demand side of family planning and, more generally, the reproductive health and well-being of their communities.

Although studies have assessed the role of religious beliefs in shaping contraceptive adoption and family formation,^25^^–^^30^ there are gaps in evidence regarding the roles of religious leaders in influencing contraceptive uptake. To that end, given the implication of religious beliefs for contraceptive adoption, this article documents the Nigerian Urban Reproductive Health Initiative's (NURHI's) activities partnering with religious leaders to promote contraceptive use in Nigeria and attempts to explore the association between exposure to family planning messages from religious leaders and contraceptive uptake in selected locations in the country.

### Religious Beliefs and Contraceptive Adoption: Theoretical Perspectives

The conceptualization of religious influence on demographic outcomes dates back to the early work of Goldsheider in 1971.^29^^,^^31^ Goldscheider^32^ posited 3 hypotheses—the characteristics hypothesis, the minority ground hypothesis, and the particularized theology hypothesis—to explain the influence of religion. As demonstrated in the works of Hirsch^31^ and Agadjanian and Yabiku,^29^ the characteristics hypothesis states that the influence of religion is only attributable to underlying factors, such as socioeconomic characteristics. The minority hypothesis suggests that family formation is essentially influenced by an individual's fertility desires to ensure the preservation of status by minority religious groups within the society. Lastly, the particularized theology hypothesizes that religious influence on demographic outcomes are attributable to doctrines or theological differences of various religions.

Our study builds on the particularized theology hypothesis, which suggests that religious belief itself is subject to underlying contextual influences and teachings, therefore giving importance to religious leaders and their doctrinal teachings as essential elements in contraceptive uptake and family formation processes. A study by Agadjanian^25^ lends credence to this proposition by arguing that the relationship between religious beliefs and contraceptive uptake is influenced by context-specific doctrines and concerns. Similarly, a study on the religion–fertility nexus partly implicated religious doctrinal differences for variations in fertility levels across denominational affiliations.^29^ These studies suggest that religious beliefs and doctrinal practices can greatly influence fertility decisions and behaviors.

This study is also supported by the ideation model advanced by Kincaid in 2000 (Figure).^33^ The ideation model posits that individuals hold different ideas and views about a behavior or outcome. Kincaid argues that ideation is composed of 3 domains: emotional (knowledge, values, norms, and perceived risk), cognitive (self-efficacy, preferences, and emotional response), and social interactions (social support, social influence, and interpersonal communication).^34^ Evidence has shown that the various domains of ideation have influence on contraceptive uptake.^8^^,^^35^ For example, social interactions contribute largely to shaping people's ideas and views,^33^^,^^34^ which, in turn, influence demographic behaviors such as contraceptive use and fertility.

**FIGURE:**
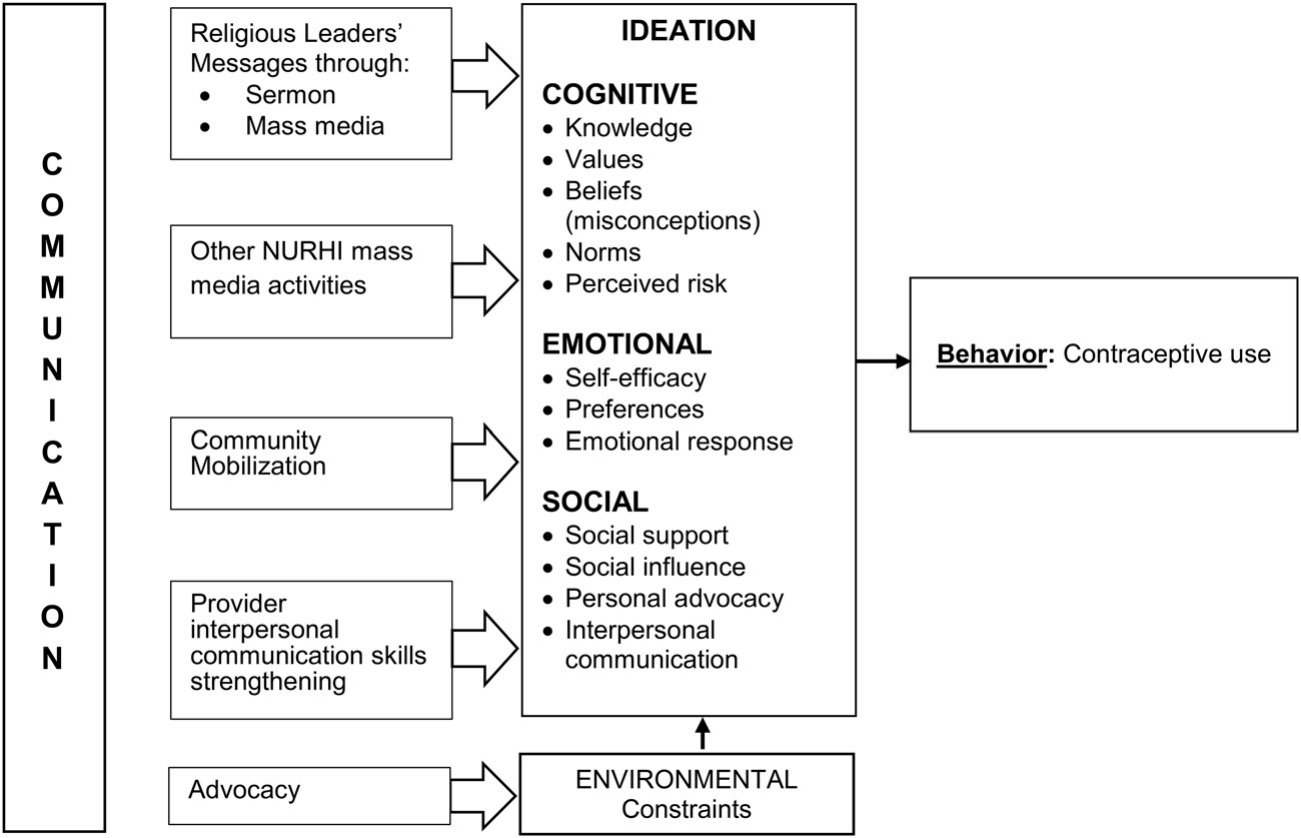
Conceptual Framework Showing Relationship Between NURHI Interventions and Contraceptive Use Abbreviation: NURHI, Nigerian Urban Reproductive Health Initiative. Adapted from Kincaid (2000) and Babalola et al. (2015).^8^

Based on the above theoretical models, individuals' beliefs, ideas, and views related to contraceptive adoption can be shaped, to a large extent, by the religious and doctrinal teachings to which they are exposed. Situating our study within this framework, we hypothesize that family planning messages that are passed through religious leaders to adherents of different faiths could help shape their ideation related to family planning and thereby contribute to increased contraceptive uptake.

## INTERVENTION

The first phase of the NURHI project started in 2009 and ran through September 2015. The second phase of the project began in October 2015 and will continue through 2020. Funded by the Bill & Melinda Gates Foundation, the primary aim of the first phase of NURHI project was to increase modern contraceptive method use, with the main focus on the urban poor in selected Nigerian cities. Currently, about 48.6% of Nigeria's population lives in urban areas; and, estimates suggest that by 2050, this will exceed 50%.^36^ Evidence shows that the urban poor tend to have more children than the urban non-poor,^37^ particularly because they have limited or no access to family planning services.

The NURHI model aimed at increasing contraceptive uptake through 3 major components—advocacy, demand creation, and service delivery. A fourth component, monitoring and evaluation, was included as a crosscutting issue. The first year of the project was devoted to formative and baseline research to identify local nuances and to understand what approaches would likely work in the different settings.

The overall goal of the project was to achieve a 20% increase in mCPR over the first 5 years of the project. To achieve this result, the project implementers identified key audience groups and influential people, such as religious and community/traditional leaders, exploring the knowledge, views, ideas, and attitudes that these audiences hold about family planning. These leaders hold crucial positions in Nigeria's contemporary society. They are important gatekeepers who, by their own statements, can affect the outcome of NURHI activities. Religious leaders, in particular, can substantially influence and shape people's ideas and views about issues such as contraceptive adoption and family formation.^29^ Their influence is particularly great in northern Nigeria because they serve as both religious and traditional leaders. For instance, the sultan of Sokoto is both a traditional ruler and the head of Islamic movement in Nigeria.

In order to achieve successful results from advocacy visits with eminent leaders, the project team identified and prepared important advocacy documents to guide the dialoging activities. The documents included a booklet entitled *Reproductive Health Issues in Nigeria: The Islamic Perspectives*,^38^ Nigeria's *National Reproductive Health Strategic Framework and Plan, 2002–2006*,^39^ and the NURHI Toolkit advocacy tools.^40^ Copies of these key documents were distributed to support dialogues with religious leaders and, later, community members. Similarly, NURHI used perspectives from Islamic and Christian scriptures to engage religious leaders and discuss family planning issues. These included a biblical injunction that reads:

*But if anyone does not provide for his own, and especially for those of his household, he has denied the faith and is worse than an unbeliever*. (1 Timothy Chapter 5 Verse 8)

A relevant message from the Quran included in the handbook reads:

*… The duty of feeding and clothing nursing mothers in a seemly manner is upon the father of the child* … (Quran, Chapter 2, Verse 233)

Both scriptures are interpreted as injunctions that stipulate the need for child spacing/family planning.

The project set up an advocacy core group (ACG) in each state. The members of the ACG were identified through stakeholder mapping exercises. The ACG included representatives of the media, market women, professional women, technocrats, and notable community members. In all of the project cities, ACG members were trained in advocacy skills and were involved in developing advocacy strategies. They were also given specific instructions/trainings on how to dialogue with religious leaders of the main faith groups in Nigeria—Christianity and Islam. Selected from within the community, ACG members were regarded as the community voice. The project worked through them to reach the community/religious leaders first and then the community at large. Advocacy visits were used as the entry point to reach religious and community/traditional leaders and gain access to and trust within communities. During the various advocacy visits, the project implementers advocated for the health benefits of family planning, such as a reduction in maternal and newborn morbidity and mortality as well as improved health and quality of life for families.

The ACGs also met with other groups, including community members, policy makers, local government chairmen, and technocrats. Together, they successfully worked with religious leaders to give statements in support of family planning in public gatherings and on the media. Through these advocacy activities, eminent leaders, such as the emirs of Ilorin, Kano, and Zaria, made statements in support of family planning, which were repeatedly aired on radio and television.

In some parts of northern Nigeria, contraception is viewed negatively as an instrument or a ploy to depopulate the Muslim population. Anticipating a strong negative reaction, the interventions were carefully planned and implemented in a religiously and culturally sensitive manner. The religious and community-based messaging emphasized the health benefits of childbirth spacing, including the reduction of maternal and child mortality and an improved quality of life for families and the nation at large. Moreover, while working with religious leaders using the book *Reproductive Health Issues in Nigeria: The Islamic Perspectives,* the Christian community requested their own handbook. This led to the production of a Christian advocacy handbook entitled *Christian Perspectives on Reproductive Health and Family Planning in Nigeria*,^41^ which was launched by Nigeria's Minister of Health at the 4th National Family Planning Conference in 2016. The document has since been adopted by the Christian community and is being used extensively to promote family planning. NURHI also provided technical support toward updating the Islamic perspectives handbook.

Other small advocacy handbooks developed by NURHI included those with Christian and Islamic sermon notes for family planning. The handbooks guide Christian and Islamic clerics/leaders as they advocate for the health and social benefits of family planning during their services, using scriptural perspectives for their messaging.

In addition to receiving messages through the religious leaders' interventions, community members were exposed to community-based media activities that focused on behavior change, improved availability and access to family planning, and improved quality of family planning services. Evidence from a NURHI intervention to improve service provision is available elsewhere.^42^

## METHODS

### Study Design and Participants

First, to document NURHI activities using religious leaders to promote contraceptive uptake in Nigeria, the project's advocacy and research staff provided relevant information on the design and implementation of intervention activities. Additional information was obtained from a review of NURHI program documents.

Second, to assess association between exposure to family planning messages from religious leaders and contraceptive use, the study used cross-sectional data from a survey conducted by the Measurement, Learning and Evaluation (MLE) project in 2015 among a randomly selected sample of women of reproductive age (15 to 49) in 4 selected Nigerian states—Federal Capital Territory, Kaduna, Kwara, and Oyo (N=10,713). The survey was conducted as part of an endline evaluation of the effects of the first phase of the NURHI project in selected states in Nigeria. Evidence from the 2013 Nigeria Demographic and Health Survey^1^ and recent analysis by Adeyanju and colleagues^43^ showed an increase in the mCPR in these targeted locations.

A 2-stage sampling selection process was employed to select respondents for the survey. In the first stage, clusters were randomly selected in each of the selected study locations, with the number of selected clusters proportional to the size of each location. In the second stage, all the dwelling units and households within the dwelling units in selected clusters were listed, and random selection resulted in a representative sample of 41 households per cluster. Eligible respondents—women aged 15 to 49 who gave consent to participate—within the selected households were interviewed face to face using a semistructured questionnaire. The present study excluded women who reported not using contraceptives because they were pregnant. The analytic sample for the study was 9,725.

### Variables Measurement

The outcome variable analyzed in this study is current use of a modern contraceptive method, defined as currently using a modern method, coded as ‘1,’ or not currently using a modern method, coded as ‘0.’ The key explanatory variable for the study is exposure to family planning messages from religious leaders. The operational definitions for this and other selected covariates are presented in Table 1. The selection of these variables was guided by the reviewed literature and the theoretical models.

**TABLE 1.** Independent Variables for Modeling Women's Uptake of Modern Contraceptive Methods in Selected Nigerian States

Variables	Operational Definitions
Exposure to family planning message	Self-reported exposure to family planning message from religious leaders, categorized as “0” had no exposure or “1” had exposure. This variable was derived from the question: “In the past year, have you heard or seen a religious leader speaking publicly in favour of family planning/child birth spacing?”
Age of respondent	Self-reported age of respondent at time of survey, categorized as: 15–24, 25–34, 35+
Religion	Respondents' religions: Catholic, Other Christian, Muslim
Parity	Number of children ever born: 0, 1–2, 3–4, 5+
Education	Highest level of education attained: none, primary, secondary, post-secondary
State of residence	Current state of residence: Federal Capital Territory, Kwara, Kaduna, Oyo
Ethnic affiliation	Respondents' ethnic affiliation: Hausa/Fulani, Igbo, Yoruba, other
Current marital status	Marital status at time of survey: married/cohabiting, never married, previously married
Wealth index	Composite index of household items/amenities, electrical appliances, toilet facility, drinking water, and floor/wall materials grouped into a quintile: (1) poorest, (2) poorer, (3) middle, (4) richer, (5) richest
Fertility desire	Respondents' desire to have another child: (1) want another child, (2) does not want another child
Need anyone's permission to use family planning	Respondent's need for someone's permission before use of family planning, categorized as: (1) Yes, permission of someone needed, (2) No, permission not needed. This variable captures a situation where a woman requires the permission of her husband/partner, mother-in-law, or someone else, before she can use a family planning method
Perceived self-efficacy	Perceived self-efficacy about family planning, generated as a composite score variable from responses to Likert-scale questions on women's level of agreement with the ideation statements. These were categorized into a tertile as: (1) low, (2) medium, (3) high; Cronbach's alpha was 0.89
Acceptance of myths and misconceptions about contraceptives	Acceptance of myths and misconceptions about contraceptives, generated as a composite score variable from responses to Likert-scale questions on women's level of agreement with the ideation statements. These were categorized into a tertile as: (1) low, (2) medium, (3) high. Cronbach's alpha was 0.89
Other NURHI interventions	Other NURHI interventions numbering 27. An overall index, i.e., composite scores, was generated to reflect the extent of exposures that respondents had to the various activities. The composite variable was categorized into a tertile as: (1) low exposure, (2) medium exposure, (3) high exposure. Cronbach's alpha for the 27-item additive index was 0.90

Abbreviation: NURHI, Nigerian Urban Reproductive Health Initiative.

### Statistical Analysis

Bivariate and multivariable analytical approaches were employed to explore significant relationships. Given the dichotomous nature of the outcome variable, we undertook binary logistic regression analysis in the multivariable analysis. Four models were fitted in the analysis. Model 1 presents the results of unadjusted analysis that examined the association of exposure to family planning messages from religious leaders with contraceptive use. Model 2 adjusted for selected background characteristics, including religiosity, age, marital status, education, ethnicity, wealth status, children ever born, state of residence, and fertility desire. Evidence from the reviewed literature established these variables as important predictors of contraceptive use. In addition to the variables included in Model 2, we also adjusted for women's exposure to other NURHI interventions in Model 3. Finally, Model 4 is the full model into which we incorporated all of the explanatory variables, including contraceptive ideational variables of perceived self-efficacy about family planning uptake and myths and misconceptions about family planning.

Results from the analysis were presented as odds ratios (ORs) and 95% confidence intervals (CIs). All analysis was done using Stata version 13.0 (StataCorp LLC, College Station, TX, USA).

## RESULTS

### Outcomes of NURHI Advocacy Visits

Information provided by NURHI staff and evidence from the review of program documents indicated that the project's advocacy visits to religious and traditional leaders recorded many successes. For instance, after the advocacy visit to the Emir of Kano, he fully supported maternal and child health, including family planning, reduction or elimination of gender-based violence, improvement in girl-child education, and small family formation, particularly among the poor. The Emir has continued to give inspiring statements in support of family planning in public gatherings and through the media. Although other activities or interventions may have influenced contraceptive uptake in the study locations, the open discussions about family planning at various public events by influential traditional/religious leaders immediately after advocacy visits suggests the usefulness and significance of this program.

Although the advocacy work is ongoing, previously implemented activities in the project locations in the north and south had supported a growing culture of open discussion about family planning in public spaces. Following the NURHI interventions, the level of knowledge that the primary aim of family planning is not to control population but to save lives increased among many religious leaders. Evidence suggests that in addition to family planning being discussed freely and openly in the public places, which was not the case many years ago, many religious women are beginning to see that child spacing and using contraception are not sinful.

NURHI's advocacy work to increase family planning uptake has been largely viewed as successful. Adeyanju et al's^43^ findings support this claim by documenting that the NURHI project contributed to the increase in modern contraceptive use in Nigeria during the post-project period.

### Descriptive Results

The highest percentages of respondents were currently married or cohabiting (65.4%), Muslim (54.3%), from households in the richest wealth quintile (38.7%), aged 15 to 24 years (37%), residents of Kaduna state (52.1%), of Hausa/Fulani ethnic origin (31.4%), and had a secondary education (45.2%) (Table 2). More than 99% of the respondents were religious, with 72.9% reporting that they were strongly religious. The analysis also indicates an average parity of 2.8, with the majority of women reporting their desire to have another child (78.9%) and the need to gain someone's permission to use family planning (86.4%). Results also showed that the decision of majority (66.4%) of the women to use family planning was influenced by religion, whereas only one-third (33.6%) reported that their decision to use contraceptives was never influenced by religion. About 2 in 5 women reported having exposure to family planning messages from religious leaders in the past year.

**TABLE 2.** Background Characteristics of Study Participants and Other Selected Variables, Selected Nigerian States, 2015 (N=9,725)

Characteristics	Value
Religion, %	
Catholic	6.0
Other Christian	39.7
Muslim	54.3
Extent of religiosity, %	
Strongly religious	72.9
Somewhat religious	27.1
Current age, years, %	
15–24	37.0
25–34	32.7
35+	30.3
Current age, years, mean	28.9
Education, %	
None	9.5
Primary	24.6
Secondary	45.2
Post-secondary	20.7
Parity, mean	2.8
City of residence, %	
FCT	14.5
Kaduna	52.1
Kwara	10.6
Oyo	22.9
Ethnic affiliation, %	
Hausa/Fulani	31.4
Igbo	6.2
Yoruba	32.5
Others	29.9
Current marital status, %	
Married/cohabiting	65.4
Never married	30.1
Previously married	4.5
Wealth index, %	
Poorest	9.8
Poorer	10.3
Middle	15.7
Richer	25.6
Richest	38.7
Fertility desire, %	
Want another child	78.9
Want no more	21.1
Need anyone's permission to use FP, %	
Yes	86.4
No	13.6
Degree to which religion influences FP decision, %	
Never	33.6
Somewhat	30.8
Frequent/always	35.6
Had exposure to religious leaders' message in favor of FP, %	
No	60.2
Yes	39.8

Abbreviations: FCT, Federal Capitol Territory; FP, family planning.

### Bivariate Analysis

Table 3 presents the results of bivariate relationship between modern contraceptive uptake and selected characteristics. The results show that contraceptive uptake varied significantly by all of the selected characteristics (*P*<.001). Compared with their counterparts, contraceptive use was higher among Christians (31.3%), somewhat religious respondents (25.8%), those who had exposure to religious leaders' message in favor of family planning (30.0%), respondents aged 25 to 34 (32.6%), women who had tertiary education (31.9%), married women (29.8%), women from households in richer and richest wealth categories (28%), and women who desired no more children (40.1%). The results further reveal a higher uptake of contraceptives among women with a high self-efficacy score (38.6%) and those with high exposure to different NURHI interventions (35.5%), compared with respondents in the low or medium categories (self-efficacy: 7.5% and 26.8%, respectively; exposure to other NURHI interventions: 14.5% and 24.5%, respectively). Conversely, contraceptive use was lowest among respondents with a high score for acceptance of myths and misconceptions about family planning (16.9%) compared with those with a medium or low score.

**TABLE 3:** Percentage Distribution of Respondents According to Contraceptive Use and Selected Characteristics, Selected Nigerian States, 2015

Characteristics	Currently Using Modern Method (%)	Not Using Modern Method (%)	Chi-Square
Religion			208.0
Catholic	24.5	75.5	
Other Christian	31.3	68.7	
Muslim	18.2	81.8	
Religiosity			8.42
Strongly religious	22.9	77.1	
Somewhat religious	25.8	74.2	
Had exposure to religious leaders' message in favor of FP			120.0
No	20.0	80.0	
Yes	30.0	70.0	
Current age, years			542.0
15–24	10.6	89.4	
25–34	32.6	67.4	
35+	30.0	70.0	
Education			129.0
None	15.4	84.6	
Primary	20.1	79.9	
Secondary	23.8	76.2	
Post-secondary	31.9	68.1	
Parity			586.0
0	9.1	90.9	
1–2	26.5	73.5	
3–4	36.8	63.2	
5+	28.4	71.6	
State of residence			135.0
FCT	28.4	71.6	
Kaduna	18.9	81.1	
Kwara	27.3	72.7	
Oyo	29.8	70.2	
Ethnic affiliation			353.0
Hausa/Fulani	12.4	87.6	
Igbo	24.5	75.5	
Yoruba	31.7	68.3	
Others	27.6	72.4	
Current marital status			400.0
Married/cohabiting	29.8	70.2	
Never married	11.2	88.8	
Previously married	13.8	86.2	
Wealth index			235.0
Poorest	9.9	90.1	
Poorer	13.4	86.6	
Middle	23.6	76.4	
Richer	28.3	71.7	
Richest	27.7	72.3	
Fertility desire			382.0
Want another child	19.0	81.0	
Does not want another child	40.1	59.9	
Need anyone's permission to use FP			6.2
Yes	23.7	76.3	
No	26.9	73.1	
Self-efficacy and FP			926.0
Low	7.5	92.5	
Medium	26.8	73.2	
High	38.6	61.4	
Acceptance of myths and misconception index on FP			126.0
Low	27.9	72.1	
Medium	25.7	74.3	
High	16.9	83.3	
Exposure to other NURHI interventions			391.0
Low exposure	14.5	85.5	
Medium exposure	24.5	75.5	
High exposure	35.5	64.5	

Abbreviations: FCT, Federal Capitol Territory; FP, family planning; NURHI, Nigerian Urban Reproductive Health Initiative.

### Multivariable Analysis

Results from logistic regression analysis examining the influence of key independent variable and other selected characteristics are presented in Table 4. In the unadjusted model, contraceptive uptake among women who had exposure to family planning messages from religious leaders was significantly higher than among those who had no exposure (OR=1.70; 95% CI, 1.54 to 1.87; *P*<.001) (Table 4). After adjusting for the effects of selected background characteristics in Model 2, similar findings were obtained (OR=1.33; 95% CI, 1.17 to 1.51; *P*<.001).

**TABLE 4.** Relationship Between Current Use of Modern Contraception and Exposure to Family Planning Messages From Religious Leaders, Selected Nigerian States, 2015

Characteristics	Model 1^a^ OR (95% CI)	Model 2^b^ OR (95% CI)	Model 3^c^ OR (95% CI)	Model 4^d^ OR (95% CI)
Had exposure to religious leader's message in favor of FP				
No	1.00	1.00	1.00	1.00
Yes	1.70 (1.54, 1.87)***	1.33 (1.17, 1.51)***	1.27 (1.12, 1.45)***	1.13 (0.99, 1.29)
Religiosity				
Strongly religious		1.00	1.00	1.00
Somewhat religious		1.30 (1.12, 1.50)***	1.31 (1.13, 1.52)***	1.27 (1.09, 1.48)**
Age, years				
15–24		1.00	1.00	1.00
25–34		1.73 (1.40, 2.14)***	1.67 (1.36, 2.06)***	1.62 (1.31, 2.00)***
35+		0.90 (0.70, 1.17)	0.86 (0.67, 1.12)	0.87 (0.67, 1.14)
Current marital status				
Married/cohabiting		1.00	1.00	1.00
Never married		1.61 (0.26, 0.39)**	1.54 (1.10, 2.18)*	1.42 (0.99, 2.03)
Previously married		0.27 (0.18, 0.39)***	0.25 (0.17, 0.37)***	0.24 (0.16, 0.35)***
Education				
None		1.00	1.00	1.00
Less than secondary		1.15 (0.87, 1.53)	1.07 (0.81, 1.42)	0.99 (0.74, 1.32)
Secondary		1.49 (1.12, 1.98)**	1.36 (1.02, 1.81)*	1.15 (0.86, 1.55)
Post-secondary		1.98 (1.43, 2.74)***	1.76 (1.27, 2.43)**	1.38 (0.99, 1.93)
Ethnic affiliation				
Hausa/Fulani		1.00	1.00	1.00
Igbo		1.57 (1.14, 2.17)**	1.62 (1.17, 2.24)**	1.38 (1.00, 1.92)
Yoruba		2.42 (1.90, 3.08)***	2.33 (1.82, 2.98)***	1.95 (1.51, 2.51)***
Others		2.52 (2.09, 3.04)***	2.53 (2.09, 3.05)***	2.10 (1.73, 2.56)***
State of residence				
Kaduna		1.00	1.00	1.00
FCT		0.94 (0.77, 1.15)	0.96 (0.78, 1.19)	1.03 (0.84, 1.28)
Kwara		0.74 (0.57, 0.97)*	0.62 (0.47, 0.83)**	0.84 (0.63, 1.13)
Oyo		1.12 (0.91, 1.39)	1.01 (0.81, 1.25)	1.04 (0.83, 1.32)
Wealth index				
Poorest		1.00	1.00	1.00
Poorer		1.39 (0.98, 1.95)	1.32 (0.94, 1.85)	1.24 (0.86, 1.77)
Middle		2.62 (1.91, 3.59)***	2.35 (1.71, 3.23)***	2.09 (1.52, 2.89)***
Richer		3.41 (2.50, 4.66)***	2.83 (2.06, 3.87)***	2.57 (1.86, 3.55)***
Richest		2.78 (2.00, 3.86)***	2.27 (1.63, 3.16)***	2.04 (1.46, 2.87)***
Parity				
0		1.00	1.00	1.00
1–2		5.50 (3.90, 7.69)***	5.01 (3.56, 7.05)***	4.86 (3.41, 6.92)***
3–4		7.93 (5.46, 11.53)***	7.34 (5.04, 10.68)***	7.00 (4.73, 10.35)***
5+		8.51 (5.72, 12.65)***	7.98 (5.35, 11.86)***	7.34 (4.84, 11.12)***
Fertility desire				
Want another child		1.00	1.00	1.00
Want no more		1.99 (1.67, 2.37)***	1.93 (1.62, 2.30)***	1.69 (1.41, 2.03)***
Needs anyone's permission to use FP				
Yes			1.00	1.00
No			1.16 (0.97, 1.40)	1.40 (1.16, 1.70)**
Exposure to other NURHI interventions				
Low exposure			1.00	1.00
Medium exposure			1.41 (1.19, 1.67)***	1.18 (0.99, 1.40)
High exposure			1.72 (1.42, 2.08)***	1.28 (1.05, 1.57)*
Perceived self-efficacy about FP use				
Low				1.00
Medium				3.48 (2.85, 4.24)***
High				4.78 (3.93, 5.82)***
Acceptance of myths and misconceptions about FP index				
Low				1.00
Medium				0.98 (0.84, 1.13)
High				0.58 (0.49, 0.69)***

Abbreviations: CI, confidence interval; FCT, Federal Capitol Territory; FP, family planning; NURHI, Nigerian Urban Reproductive Health Initiative; OR, odds ratio.

* *P*<0.05;

** *P*<0.01;

*** *P*<0.001.

a Model 1=unadjusted analysis.

b Model 2=adjusted for selected background characteristics, including religiosity, age, marital status, education, ethnicity, wealth status, parity, state of residence, and fertility desire.

c Model 3=adjusted for selected background characteristics, including religiosity, age, marital status, education, ethnicity, wealth status, parity, state of residence, and fertility desire, and for women's exposure to other NURHI interventions.

d Model 4=adjusted for all variables included in Model 3, plus perceived self-efficacy about family planning uptake and myths and misconceptions about family planning index.

The significant association of exposure to family planning messages from religious leaders with contraceptive uptake was reported again in Model 3, which adjusted for additional variables. The influence of exposure to family planning messages from religious leaders became statistically insignificant with the introduction of ideational variables in Model 4 (OR=1.13; 95% CI, 0.99 to1.29). Summarizing the results from all model specifications, we found a statistically significant influence of exposure to family planning messages from religious leaders in promoting contraceptive uptake, with or without exposure to other NURHI interventions and irrespective of respondents' background characteristics and ideation variables—with the exception of perceived self-efficacy about family planning.

Some control variables included in the full model were also found to be significantly associated with contraceptive use. For instance, desire for no more children (OR=1.69; 95% CI, 1.41 to 2.00; *P*<.01), being aged 25 to 34 (OR=1.62; 95% CI, 1.31 to 2.00; *P*<.001), being a Yoruba woman (OR=1.90; 95% CI, 1.51 to 2.51; *P*<.001), and being from a rich household (*P*<.001) were significantly associated with contraceptive uptake, relative to those in the corresponding reference categories. Similarly, composite variables on other NURHI interventions, religiosity, number of children ever born, permission to use family planning, contraceptive ideations about myths/misconceptions, and perceived self-efficacy were significantly associated with contraceptive use (*P*<.05).

## DISCUSSION

In a religiously pluralistic Nigerian setting, decision-making processes in different areas of and groups within the country are largely influenced by religious beliefs and practices.^13^^,^^44^ Consequently, religion is firmly intertwined with the day-to-day life of an average Nigerian. Considering the importance of religion to a majority of the population as well as the influential positions occupied by religious leaders in Nigeria, the NURHI project adopted strategies to increase contraceptive uptake by engaging religious leaders in advocacy work. Having successfully implemented this project in selected locations in the country, the present analysis explored the association between exposure to religious leaders' family planning messages and modern contraceptive uptake in Nigeria. Despite the key position occupied by religious leaders in the country, their potential role in promoting contraceptive use had not been adequately explored until now.

Our findings established that the majority of respondents considered themselves strongly religious. The concept of religiosity connotes a strong adherence to religious beliefs and doctrinal teachings. Thus, this result indicates that the lives and behaviors of a high proportion of respondents across different societal strata are strongly influenced by religious beliefs. A previous study had also found religion to be a well-entrenched factor that influenced decision-making processes across different settings in Nigeria.^23^

This study also revealed that the family planning decisions made by the majority of women were influenced by religion. This demonstrates the importance of addressing religion in order to increase contraceptive use in Nigeria. Existing literature confirms the key role religion plays in shaping the decision to use contraceptive methods.^26^^,^^27^^,^^30^ Our findings show that the NURHI intervention that engaged religious leaders in promoting family planning had substantial coverage, as a large proportion of women were exposed to religious leaders' messages on family planning in the past year. Of the women who had this exposure, about one-third were currently using modern contraceptive methods. Moreover, results from our multivariable analysis established a significant association between exposure to family planning messages from religious leaders and modern contraceptive uptake. The importance of exposure to religious leaders' family planning messages was further supported in the adjusted model that included background characteristics and exposure to other NURHI interventions. The fact that exposure to religious leaders' messages became insignificant in the model that included the ideational variables is not surprising and suggests that the effect of exposure to communication operates through its effect on ideation. These results have important policy implications.

First, given the importance of religion in Nigeria's sociocultural fabric^23^^,^^44^ and the position of influence, authority, and respect occupied by various religious leaders (i.e., bishops, pastors, evangelists, imams, and sheiks as well as eminent trado-religious leaders, like emirs and sultans), one way of achieving a rapid increase in Nigeria's mCPR may be to continuously engage religious leaders at all levels in advocacy efforts. Generally, Nigerians are very religious and seem to accord their religious leaders greater honor and veneration than what they confer on political leaders. As a result, religious leaders wield great influence over their large congregations. Their body language and messages can inhibit or facilitate effective health care-seeking behaviors. For example, religious belief, a key determinant of child immunization completeness,^45^ has previously been cited as an underlying factor for calling for a boycott of childhood immunization by trado-religious leaders in some parts of northern Nigeria. By engaging religious leaders in using appropriate family planning messaging, the mCPR in Nigeria will likely increase.

Second, given the established relationship between effective contraception and maternal and child health,^10^ religious leaders have the power to promote family health and well-being and contribute to the discourse and strategies on maternal and newborn morbidity and mortality reduction through congregational advocacy messages on the health benefits of family planning. Religious prohibition of contraceptive adoption still persists in Nigeria,^18^ partly because of the spread of myths and misconceptions about family planning.^21^^,^^22^ Because of this, the strategy of working with religious leaders to increase their knowledge of family planning and its benefits and, ultimately, engage them as change agents, may be crucial to increasing family planning adoption and promoting family health in Nigeria.

Moreover, our analysis indicates higher contraceptive uptake among somewhat religious women than their strongly religious counterparts. This suggests that strong adherence to religious doctrines and practices, combined with religious leaders who do not incorporate appropriate family planning messages into their communications, likely contributes to low contraceptive uptake. Religion has been established as one of the most important determinants of behaviors, including health seeking.^46^^,^^47^ As religious beliefs continue to hinder contraceptive uptake in Nigeria, engaging religious leaders as potential change agents is crucial for creating positive change. The findings from this study, therefore, underscore the importance of enlisting religious leaders in efforts to increase contraceptive uptake in Nigeria.

After including the ideational variable of self-efficacy in our analytical model, the relationship between family planning messages from religious leaders and contraceptive uptake became insignificant. As established in a previous study,^8^ this finding suggests the importance of incorporating contraceptive ideation and communication interventions aimed at ideational variables related to contraceptive use in efforts to increase contraceptive uptake.

In addition, and as established by prior studies,^10^^,^^27^^,^^35^^,^^48^ this study documented other important predictors of contraceptive use, including fertility desire, parity, respondent's age, ethnic affiliation, wealth index, religiosity, permission to use family planning, and contraceptive ideations. Our results also underscored that other NURHI intervention activities were important for increasing contraceptive uptake in Nigeria. This finding suggests that this family planning intervention, if as well executed as other NURHI activities, may yield impactful results in Nigeria and other sub-Saharan African countries with similar contexts.

### Limitations

This study is not without some limitations. Because of the nature of cross-sectional studies, we could not establish a direct cause–effect relationship. As the study was based on self-reported data, respondents may have answered questions with the aim of pleasing the interviewer, thus adding social desirability bias to the results. However, steps were taken during fieldwork to minimize bias through appropriate interviewing practices that would ensure the anonymity and confidentiality of solicited responses. It would be interesting to explore the dose–response between increased exposure to family planning messages from religious leaders and contraceptive use; however, the data do not support this type of analysis. Notwithstanding the study limitations, this article has helped to address an important gap in public health literature on the role of religious leaders in promoting contraceptive use.

## CONCLUSION

Given the high level of influence held by religious leaders in Nigeria's sociopolitical landscape, interventions that engage clerics of different faiths as change agents for shaping norms and influencing behaviors related to family planning and contraceptive use are crucial for increasing contraceptive uptake in the country. By sharing tailored scriptural messages that address important health and behavior change information to support positive family health behaviors, religious leaders create a supportive environment for women and their partners to make healthy family planning decisions for themselves and their families.

## Notes

**Peer Reviewed**

**Funding:** Bill & Melinda Gates Foundation.

## References

[1] National Population Commission (NPC) and ICF International. Nigeria Demographic and Health Survey 2013. Abuja, Nigeria, and Rockville, MD, USA: NPC and ICF International; 2014. https://dhsprogram.com/pubs/pdf/fr293/fr293.pdf. Accessed August 3, 2018.

[2] National Institute of Statistics of Rwanda (NISR), Ministry of Health (MOH), ICF International. Rwanda Demographic and Health Survey 2010. Calverton, MD: NISR, MOH, and ICF International; 2012. https://dhsprogram.com/pubs/pdf/FR259/FR259.pdf. Accessed August 3, 2018.

[3] National Statistical Office (NSO) and ICF. Malawi Demographic and Health Survey 2015–16. Zomba, Malawi, and Rockville, MD: NSO and ICF; 2017. https://dhsprogram.com/pubs/pdf/FR319/FR319.pdf. Accessed August 3, 2018.

[4] Izugbara CO Wekesah FM Adedini SA. Maternal Health in Nigeria: A Situation Update. Nairobi, Kenya: African Population and Health Research Center; 2016. http://aphrc.org/post/publications/maternal-health-nigeria-situation-update. Accessed August 3, 2018.

[5] Adeleke D. Reproductive Health and Family Planning: A Christian Perspective. A Booklet Prepared for Nigerian Urban Reproductive Health Initiative (NURHI). Abuja, Nigeria: NURHI; 2016.

[6] Akinyemi A Adedini S Hounton S. Contraceptive use and distribution of high-risk births in Nigeria: a sub-national analysis. Glob Health Action. 2015;8:29745. 10.3402/gha.v8.29745. 26562145 PMC4642363

[7] Adedini SA Odimegwu C Imasiku ENS Ononokpono DN. Unmet need for family planning: implication for under-five mortality in Nigeria. J Health Popul Nutr. 2015;33(1):187–206. 25995735 PMC4438662

[8] Babalola S John N Ajao B Speizer I. Ideation and intention to use contraceptives in Kenya and Nigeria. Demogr Res. 2015;33:211–238. 10.4054/DemRes.2015.33.8 31303859 PMC6625811

[9] Bahamondes L Valeria Bahamondes M Shulman LP. Non-contraceptive benefits of hormonal and intrauterine reversible contraceptive methods. Hum Reprod Update. 2015;21(5):640–651. 10.1093/humupd/dmv023. 26037216

[10] Cleland J Conde-Agudelo A Peterson H Ross J Tsui A. Contraception and health. Lancet. 2012;380(9837):149–156. 10.1016/S0140-6736(12)60609-6. 22784533

[11] Whitworth A Stephenson R. Birth spacing, sibling rivalry and child mortality in India. Soc Sci Med. 2002;55(12):2107–2119. 10.1016/S0277-9536(02)00002-3. 12409124

[12] Wall LL. Dead mothers and injured wives: the social context of maternal morbidity and mortality among the Hausa of northern Nigeria. Stud Fam Plann. 1998;29(4):341–359. 10.2307/172248. 9919629

[13] Adedini SA Odimegwu C Imasiku ENS Ononokpono DN. Ethnic differentials in under-five mortality in Nigeria. Ethn Health. 2015;20(2):145–162. 10.1080/13557858.2014.890599. 24593689 PMC4337727

[14] Casterline JB. Determinants and Consequences of High Fertility: A Synopsis of the Evidence. Washington, DC: The World Bank; 2010. http://documents.worldbank.org/curated/en/389381468147851589/pdf/630690WP0P10870nants0pub08023010web.pdf. Accessed August 3, 2018.

[15] Ibisomi L. Is age difference between partners associated with contraceptive use among married couples in Nigeria? Int Perspect Sex Reprod Health. 2014;40(1):39–45. 10.1363/4003914. 24733060

[16] Lee J Berenson AB Patel PR. Characteristics of females who use contraception at coitarche: an analysis of the National Survey of Family Growth 2006-2010 database. J Women's Health (Larchmt). 2015;24(12):972–977. 10.1089/jwh.2015.5219. 26595506

[17] Bongaarts J Bruce J. The causes of unmet need for contraception and the social content of services. Stud Fam Plann. 1995;26(2):57–75. 10.2307/2137932. 7618196

[18] Lawani LO Iyoke CA Ezeonu PO. Contraceptive practice after surgical repair of obstetric fistula in southeast Nigeria. Int J Gynaecol Obstet. 2015;129(3):256–259. 10.1016/j.ijgo.2014.11.028. 25728480

[19] Adebowale SA Adedini SA Ibisomi LD Palamuleni ME. Differential effect of wealth quintile on modern contraceptive use and fertility: evidence from Malawian women. BMC Womens Health. 2014;14(1):40. 10.1186/1472-6874-14-40. 24602452 PMC3973841

[20] Huda FA Chowdhuri S Sirajuddin MF. Importance of appropriate counselling in reducing early discontinuation of Norplant in a northern district of Bangladesh. J Health Popul Nutr. 2014;32(1):142–148. 24847603 PMC4089082

[21] Gueye A Speizer IS Corroon M Okigbo CC. Belief in family planning myths at the individual and community levels and modern contraceptive use in urban Africa. Int Perspect Sex Reprod Health. 2015;41(4):191–199. 10.1363/intsexrephea.41.4.0191. 26871727 PMC4858446

[22] Okigbo C Speizer I Domino M Curtis S. A multilevel logit estimation of factors associated with modern contraception in urban Nigeria. World Med Health Policy. 2017;9(1):65–88. 10.1002/wmh3.215 31428512 PMC6699167

[23] Pinter B Hakim M Seidman DS Kubba A Kishen M Di Carlo C. Religion and family planning. Eur J Contracept Reprod Health Care. 2016;21(6):486–495. 10.1080/13625187.2016.1237631. 27681868

[24] Ilyas M Alam M Ahmad H Ghafoor S. Abortion and protection of the human fetus: religious and legal problems in Pakistan. Hum Reprod Genet Ethics. 2009;15(2):55–59. 10.1558/hrge.v15i2.55. 19957496

[25] Agadjanian V. Religious denomination, religious involvement, and modern contraceptive use in southern Mozambique. Stud Fam Plann. 2013;44(3):259–274. 10.1111/j.1728-4465.2013.00357.x. 24006073 PMC4604208

[26] Farrell M Masquelier A Tissot E Bertrand J. Islam, polygyny and modern contraceptive use in Francophone sub-Saharan Africa. Etude Popul Afr. 2014;28(3). 10.11564/28-3-631

[27] Prettner K Strulik H. It's a sin—contraceptive use, religious beliefs, and long-run economic development. Rev Dev Econ. 2017;21(3):543–566. 10.1111/rode.12280

[28] Gyimah SO Adjei JK Takyi BK. Religion, contraception, and method choice of married women in Ghana. J Relig Health. 2012;51(4):1359–1374. 10.1007/s10943-011-9478-4. 21567266

[29] Agadjanian V Yabiku ST. Religious affiliation and fertility in a sub-Saharan context: dynamic and lifetime perspectives. Popul Res Policy Rev. 2014;33(5):673–691. 10.1007/s11113-013-9317-2. 26500383 PMC4612361

[30] Doctor HV Phillips JF Sakeah E. The influence of changes in women's religious affiliation on contraceptive use and fertility among the Kassena-Nankana of northern Ghana. Stud Fam Plann. 2009;40(2):113–122. 10.1111/j.1728-4465.2009.00194.x. 19662803

[31] Hirsch JS. Catholics using contraceptives: religion, family planning, and interpretive agency in rural Mexico. Stud Fam Plann. 2008;39(2):93–104. 10.1111/j.1728-4465.2008.00156.x. 18678173

[32] Goldscheider C. Population, Modernization, and Social Structure. Boston: Little, Brown, and Co.; 1971.

[33] Kincaid DL. Social networks, ideation, and contraceptive behavior in Bangladesh: a longitudinal analysis. Soc Sci Med. 2000;50(2):215–231. 10.1016/S0277-9536(99)00276-2. 10619691

[34] Kincaid DL. Mass media, ideation, and behavior: a longitudinal analysis of contraceptive change in the Philippines. Communic Res. 2000;27(6):723–763. 10.1177/009365000027006003

[35] Babalola S Folda L Babayaro H. The effects of a communication program on contraceptive ideation and use among young women in northern Nigeria. Stud Fam Plann. 2008;39(3):211–220. 10.1111/j.1728-4465.2008.168.x. 18853642

[36] World Data Atlas. Nigeria - Urban population as a share of total population. Knoema Website. https://knoema.com/atlas/Nigeria/Urban-population. Accessed August 3, 2018.

[37] Beguy D Ezeh AC Mberu BU Emina JBO. Changes in use of family planning among the urban poor: evidence from Nairobi slums. Popul Dev Rev. 2017;43 (S1):216–234. 10.1111/padr.12038

[38] Pathfinder International, POLICY Project. Reproductive Health Issues in Nigeria: The Islamic Perspectives. Abuja, Nigeria: Pathfinder International Nigeria; 2004. https://berkleycenter.georgetown.edu/publications/reproductive-health-issues-in-nigeria-the-islamic-perspectives. Accessed August 3, 2018.

[39] Federal Ministry of Health (FMOH). National Reproductive Health Strategic Framework and Plan, 2002–2006. Abuja, Nigeria: FMOH; 2002. http://www.policyproject.com/pubs/countryreports/NIG_RHStrat.pdf. Accessed August 5, 2018.

[40] Nigerian Urban Reproductive Health Initiative (NURHI) Toolkit. Advocacy. NURHI Website. http://www.nurhitoolkit.org/program-areas/advocacy#.W2dqqBpKg6i. Accessed August 5, 2018.

[41] Nigerian Urban Reproductive Health Initiative (NURHI 2) Project. Christian Perspectives on Reproductive Health and Family Planning in Nigeria. Abuja, Nigeria: NURHI 2; 2017. http://146.66.97.188/∼nurhi363/images/advocacydocuments/cp_rh_fp_nigeria.pdf. Accessed August 6, 2018.

[42] Krenn S Cobb L Babalola S Odeku M Kusemiju B. Using behavior change communication to lead a comprehensive family planning program: the Nigerian Urban Reproductive Health Initiative. Glob Health Sci Pract. 2014;2(4):427–443. 10.9745/GHSP-D-14-00009. 25611477 PMC4307859

[43] Adeyanju O Tubeuf S Ensor T. The Nigerian Urban Reproductive Health Initiative: A Decomposition Analysis of the Changes in Modern Contraceptive Use (No. 1704). Abuja, Nigeria: NURHI; 2017. https://medhealth.leeds.ac.uk/download/3788/auhe_wp1704. Accessed August 14, 2018.

[44] Oguntola-Laguda D. Religion, leadership and struggle for power in Nigeria: a case study of the 2011 presidential election in Nigeria. Studia Historiae Ecclesiasticae 2015;41(2). 10.17159/2412-4265/2015/225

[45] Anyene B. Routine immunization in Nigeria: The role of politics, religion and cultural practices. Afr J Health Econ. 2014;3(1):0002. http://www.ajhe.org/uploads/55/3075_pdf.pdf.

[46] Obasohan PE. Religion, ethnicity and contraceptive use among reproductive age women in Nigeria. Int J MCH AIDS. 2015;3(1):63–73. 27621987 PMC4948172

[47] Ononokpono DN Odimegwu CO Imasiku E Adedini S. Contextual determinants of maternal health care service utilization in Nigeria. Women Health. 2013;53(7):647–668. 10.1080/03630242.2013.826319. 24093448

[48] Oye-Adeniran BA Adewole IF Umoh AV. Community-based study of contraceptive behaviour in Nigeria. Afr J Reprod Health. 2006;10(2):90–104. 10.2307/30032462. 17217121

